# Common Mechanism for Target Specificity of Protein- and DNA-Targeting ADP-Ribosyltransferases

**DOI:** 10.3390/toxins13010040

**Published:** 2021-01-07

**Authors:** Toru Yoshida, Hideaki Tsuge

**Affiliations:** 1 Faculty of Science, Japan Women’s University, 2-8-1 Mejirodai, Bunkyo-ku, Tokyo 112-8681, Japan; yoshidat@fc.jwu.ac.jp; 2 Faculty of Life Sciences, Kyoto Sangyo University, Kamigamo-motoyama, Kita-ku, Kyoto 603-8555, Japan; 3 Institute for Protein Dynamics, Kyoto Sangyo University, Kamigamo-motoyama, Kita-ku, Kyoto 603-8555, Japan; 4 Center for Molecular Research in Infectious Diseases, Kyoto Sangyo University, Kamigamo-motoyama, Kita-ku, Kyoto 603-8555, Japan

**Keywords:** ADP-ribosyltransferase, substrate recognition, target residue specificity, complex structure of enzyme and substrate

## Abstract

Many bacterial pathogens utilize ADP-ribosyltransferases (ARTs) as virulence factors. The critical aspect of ARTs is their target specificity. Each individual ART modifies a specific residue of its substrates, which could be proteins, DNA, or antibiotics. However, the mechanism underlying this specificity is poorly understood. Here, we review the substrate recognition mechanism and target residue specificity based on the available complex structures of ARTs and their substrates. We show that there are common mechanisms of target residue specificity among protein- and DNA-targeting ARTs.

## 1. Introduction

Many pathogenic bacteria produce protein toxins and effectors that covalently modify host proteins to affect their functions or mimic host protein functions to interfere with host cell regulatory processes [1]. ADP-ribosyltransferases (ARTs) are a well-known type of bacterial toxin [2]. They transfer an ADP-ribose moiety from nicotinamide adenine dinucleotide (NAD^+^) onto a target protein to generate an ADP-ribosylated protein and nicotinamide. The bulky and negatively charged ADP-ribose moiety affects the protein function by sterically blocking interactions with partner molecules, inducing conformational changes, or creating docking sites for new interaction partners [3].

**Figure 1:**
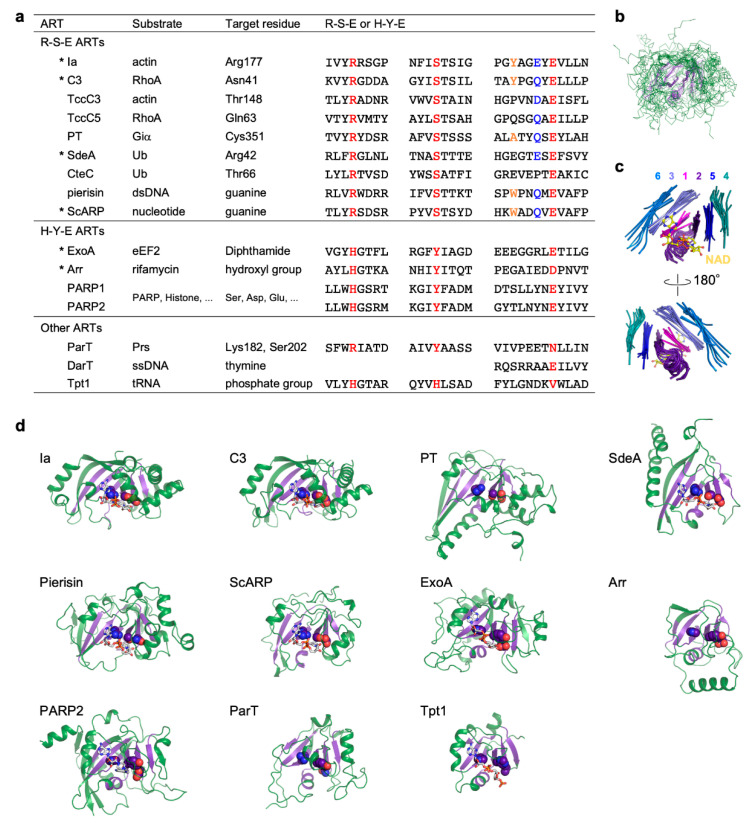
Comparison of R-S-E class, H-Y-E class, and other ADP-ribosyltransferases. (**a**) Substrate, target residue, and conserved motifs. Conserved R-S-E and H-Y-E residues are shown in red. In the R-S-E motif, the third aromatic residue and the sixth glutamate or glutamine residue in the ADP-ribosylating toxin turn-turn (ARTT) loop (X-X-ϕ-X-X-E/Q-X-E) are shown in orange and blue, respectively. ADP-ribosyltransferases (ARTs) with available substrate-complexed structures are indicated with asterisks. (**b**) Superimposed structures of the ART domains of the 11 ARTs are shown in (**d**). The structure of the ART core, including six strands and the helix, are shown in violet. (**c**) Close-up views of ART core structures. Individual strands are shown in different colors and are numbered from the N-terminus to the C-terminus. NAD bound to Ia is shown as a ball-and-stick model. (**d**) Structures of the ART domain. ART core structures are shown in violet. R-S-E and H-Y-E residues are shown as sphere models. NAD or NAD analogs are shown as stick models. Protein Data Bank (PDB) IDs: Ia, 4h03; C3, 4xsh; PT, 1bcp; SdeA, 5yij; Pierisin, 5h6j; ScARP, 5zj5; ExoA, 2zit; Arr, 2hw2; PARP2, 6tx3; ParT, 6d0h; Tpt1, 6e3a.

In addition to their function as bacterial toxins, ARTs have been implicated in a wide range of processes in organisms from all kingdoms of life, including the toxin–antitoxin system of bacteria and the DNA damage repair, transcription, cell-cycle progression, and cell division processes of eukaryotes [4]. Individual ARTs have specific substrates and modify specific residues or functional groups of not only proteins but also DNA, RNA, and antibiotics [5]. ARTs are subdivided into two classes based on their conservation of three significant motifs: the R-S-E class, which is related to cholera toxin; and the H-Y-E class, which is related to diphtheria toxin (Figure 1a) [6].

ARTs transfer an ADP-ribose moiety specifically onto nucleophilic atoms as follows: onto the oxygen atom of the carboxyl groups of aspartate and glutamate, the hydroxyl groups of serine and antibiotics, and the 5′ phosphate group of double-stranded DNA (dsDNA); onto the nitrogen atom of the amino group of the guanidino groups of arginine and guanine, the imidazole group of diphthamide, and the carboxamide groups of asparagine and glutamine; and onto the sulfur atom of the thiol group of cysteine [7]. However, the precise mechanism by which ARTs modify a specific residue or functional group remains an open question. In this review, we discuss the recognition mechanism of the target residues, including nucleophilic atoms, based on the complex structures of ARTs and their substrates.

## 2. ART Structures

Most ARTs can be subdivided into the R-S-E and H-Y-E classes, but some do not belong to either class (Figure 1a). However, superimposition of ART domains of all the ARTs shows that there is strict conservation of the core structure consisting of six strands and a helix (Figure 1b–d) [6]. The R-S-E and H-Y-E residues are located on strands of the core structure: The arginine and histidine, serine and tyrosine, and glutamate residues are located on the first, second, and fifth strands from the N-terminus, respectively. NAD binds to the cleft formed by the core structure. The arginine and histidine residues of the R-S-E and H-Y-E motifs are important for NAD binding, while the glutamate residues are essential for the cleavage of NAD^+^.

## 3. Substrates and Target Residues of R-S-E Class ARTs

Most ARTs that act as bacterial toxins belong to the R-S-E class, and many members of this class have been found to affect the actin cytoskeleton. Iota toxin (Ia) from *Clostridium perfringens* specifically modifies actin on Arg177; although the modified actin structure does not show any major conformational change compared to non-modified actin, the modified actin is not able to polymerize due to the steric hindrance from the ADP-ribose moiety, resulting in actin depolymerization and cell rounding [8]. Many Ia-homologous toxins are known: C2I from *Clostridium botulinum* [9], CDTa from *Clostridium difficile* [10], CSTa from *Clostridium spiroforme* [11], VIP2 from *Bacillus cereus* [12], and CPILEa (also known as BECa) from *C. perfringens* [13,14]. All of these toxins ADP-ribosylate Arg177 on actin [15]. C3 toxin from *Clostridium botulinum* specifically modifies Rho GTPase, the master regulator of the actin cytoskeleton, on Asn41 [16]. Rho GTPase functions as a molecular switch between active GTP-bound Rho, which transduces signals by interacting with effector proteins and inactive GDP-bound Rho. ADP-ribosylation occurs on both GTP- and GDP-bound Rho. Although the modified GTP-bound Rho is still able to bind its effector proteins [17], the modified GDP-bound Rho shows a tighter binding with guanine nucleotide dissociation inhibitor (GDI) compared to non-modified Rho, which prevents activation and results in the destruction of actin stress fibers [18]. Many C3-homologous toxins have been described, including C3lim from *Clostridium limosum* [19], C3cer from *Bacillus cereus* [20], C3stau from *Staphylococcus aureus* [21], and C3larvinA and Plx2A from *Paenibacillus larvae* [22,23]. The components of the Tc Toxin complex from *Photorhabdus luminescens*, TccC3 and TccC5, specifically modify actin on Thr148 (a different target residue than that of iota toxin) and RhoA on Gln63 (a different target residue than that of C3 toxin), respectively [24]. Because the modified residues are different, the functional consequences of TccC3 and TccC5 are different from those of iota and C3: TccC3 and TccC5 do not cause actin depolymerization but rather its polymerization or clustering. Thr148 of actin is located in the site of its interaction with thymosin-β4, which prevents actin polymerization. ADP-ribosylation of actin at Thr148 reduces the affinity for thymosin-β4 to inhibit actin sequestration and promote actin polymerization. Gln63 of RhoA is essential for the hydrolysis of GTP; the ADP-ribosylation of RhoA at Gln63 causes its persistent activation, resulting in strong stress fiber formation. Studies have also shown that ARTs are utilized as toxins for interbacterial competition [25]. Tre1 from *Serratia proteamaculans*, which is an effector protein via the type VI secretion system, specifically ADP-ribosylates Arg174 on the bacterial tubulin-like protein FtsZ. This ADP-ribosylation disrupts cell division, leading to cell elongation and cell death. Because the effector is delivered indiscriminately, *S. proteamaculans* has a means to prevent self-intoxication: The cognate immunity protein, Tri1, protects against Tre1 toxicity by physical sequestration and enzymatic ADP-ribose cleavage.

Cysteine-targeting ADP-ribosylation is unusual in ARTs, but pertussis toxin from *Bordetella pertussis* specifically modifies Cys351 on the α subunit of inhibitory trimeric G-protein (Giα), which is a conserved cysteine located in the fourth position from the C-terminus [26]. Because this ADP-ribosylation prevents the binding to G protein-coupled receptor (GPCR) due to steric hindrance, Giα remains in its inactive GDP-bound state and is unable to inhibit adenylate cyclase activity, resulting in elevation of the intracellular cAMP level. Pertussis-like toxin from *Escherichia coli* (*Ec*Plt) has been also analyzed: Rather than targeting Cys351, this toxin was found to target Lys345 and Asn347 [27].

Ubiquitin-targeting R-S-E class ARTs have been also discovered. The SidE family effector proteins from *Legionella pneumophila*—SdeA, SdeB, SdeC, and SidE—specifically modify ubiquitin (Ub) on Arg42 [28,29]. SdeA consists of N-terminal deubiquitinase (DUB), phosphodiesterase (PDE), ART, and coiled-coil (CC) domains. SdeA catalyzes ADP-ribosylation of Ub on Arg42 via its ART domain and then cleaves the pyrophosphate bond of the ADP-ribose moiety via its PDE domain to release AMP. The cleavage is coupled to a nucleophilic attack of water or a serine residue of a substrate protein, which generates phosphoribosylated Ub or a ubiquitinated protein via phosphoribose, respectively. This ubiquitination of a substrate protein is totally different from the conventional ubiquitination that proceeds through the conserved enzymes E1, E2, and E3. ADP-ribosylation and phosphoribosylation of ubiquitin prevent the reaction with E1 due to steric hindrance, which impairs several Ub-dependent processes. The type III effector, CteC, from *Chromobacterium violaceum* specifically ADP-ribosylates Thr66 of ubiquitin, which prevents the transfer of ubiquitin from E1 to E2 and thereby disrupts ubiquitin signaling [30]. CteC modifies both mono- and poly-Ub. The ADP-ribosylation of mono-Ub does not affect E1-mediated Ub activation but prevents the transfer of Ub from E1 to E2, leading to inhibition of poly-Ub chain synthesis. The ADP-ribosylation of poly-Ub prevents recognition by Ub binding domains and inhibits deubiquitinase-mediated deubiquitination, leading to dysfunction of poly-Ub chains. Two homologous proteins have been found to show the same function as CteC: CHBU from *Burkholderia ubonensis* and CHCS from *Corallococcus* show 66% and 24% sequence identity with CteC, respectively. The amino acid sequences of the CteC family members are not similar to those of other R-S-E class ARTs, and their tertiary structure has not yet been revealed.

Pierisin, which was originally purified from larvae of cabbage butterfly *Pieris rapae*, specifically modify dsDNA on the N^2^ amino group of the guanine base, causing mutations and an apoptotic response in cancer cells such as the HeLa and TMK-1 cell lines [31,32]. Pierisin may induce programmed cell death in larval cells to drive insect development, as it is found at a high level (0.4% of total protein) in the pupae of *P. rapae* [33]. It has also been speculated that pierisin functions as a defense factor against parasitization by wasps. ScARP from *Streptomyces coelicolor* can ADP-ribosylate the N^2^ amino group of the guanine bases of nucleosides and nucleotides, such as guanosine, deoxyguanosine, GTP, and deoxyGTP, whereas it cannot act against dsDNA [34]. Scabin from *Streptomyces scabies*, which is a putative virulence factor for plants such as potato, also ADP-ribosylates the N^2^ amino group of the guanine bases of nucleosides, nucleotides, single-stranded DNA, and genomic DNA [35]. Mosquitocidal toxin (MTX) from *Bacillus sphaericus*, which has 30% sequence identity with pierisin, does not target DNA but rather elongation factor Tu (EF-Tu) in *Escherichia coli*. [36]. ADP-ribosylation of EF-Tu prevents the ternary complex formation of EF-Tu, GTP, and aminoacyl-tRNA, resulting in inhibition of bacterial protein synthesis.

## 4. Substrates and Target Residues of H-Y-E Class ARTs

Exotoxin A (ExoA) from *Pseudomonas aeruginosa*, diphtheria toxin (DT) from *Corynebacterium diphtheriae*, and cholix toxin from *Vibrio cholera* specifically modify translation elongation factor (eEF2) on diphthamide699 in yeast or diphthamide715 in mammals [37,38,39]. ADP-ribosylated eEF2 is unable to mediate the translocation of peptidyl-tRNA from the A to P sites on the ribosome, inhibiting protein synthesis. Arr ADP-ribosylates the hydroxyl group of rifamycins, leading to resistance against these antibiotics [40]. Arr is widely distributed in the genomes of pathogenic and nonpathogenic bacteria. Although Arr does not show sequence similarity to known ARTs, its structure is similar to those of known ARTs. The glutamate residue in the conserved motif H-Y-E is replaced by an aspartate residue that is located on the fourth strand (rather than the fifth strand) from the N-terminus. The poly(ADP-ribose) polymerase (PARP) family comprises 17 members: PARP1 to PARP16, which includes two tankyrases (PARP5a and PARP5b) [41,42]. Poly-ADP-ribose is removed by poly(ADP-ribose) glycohydrolase (PARG) and macrodomain proteins [43]. PARPs affect various cellular processes, such as DNA repair, DNA replication, transcriptional regulation, and cell division. The best-studied PARP, PARP1, is thought to be a key player in these processes and has shown promise as a target for anti-cancer drugs.

## 5. Other ARTs

Some enzymes involved in ADP-ribosylation do not belong to the R-S-E or H-Y-E classes. ParST functions as a toxin–antitoxin (TA) system via ADP-ribosylation and is found in 18% bacterial species [44]. ParT from *Sphingobium* sp. YBL2 specifically modifies *E. coli* phosphoribosyl pyrophosphate synthetase (Prs) on Lys182 (located in the ATP binding site) or Ser202. Although Lys182 of Prs is well-conserved, *Sphingobium* sp. YBL2 has an aspartic acid, which is also known as an ADP-ribosylation target residue, at this position. ParT does not show sequence similarity with other ARTs, but its structure is similar to those of other ARTs; notably, the important residues H-Y-E are replaced by R-Y-N in ParT. ParS neutralizes ParT via protein–protein interaction. DarTG, which was originally identified from *Mycobacterium tuberculosis*, also functions in the TA system via reversible ADP-ribosylation of thymidine on single-stranded DNA (ssDNA) [45]. The ADP-ribosylation of ssDNA via DarT stalls DNA replication, and its potential toxicity can be counteracted by enzymatic removal or physical sequestration via the N-terminal macrodomain or C-terminal domain of DarG, respectively [46]. DarTs from *M. tuberculosis* and enteropathogenic *E. coli* specifically ADP-ribosylate the TNTC motif and the TTT or TCT motifs of ssDNA, respectively. Although the DarT sequence does not share sequence similarity with any other ART and its structure has not yet been revealed, DarT family sequences show conservation of the catalytic glutamate residue. Tpt1 is an essential enzyme in yeast for the final step of tRNA splicing, which generates mature tRNA from pre-tRNA [47]. Tpt1 catalyzes removal of the 2′-phosphate generated at the splice junction via a two-step reaction: (i) The 2′-phosphate of tRNA nucleophilically attacks the 1″-carbon of the ribose of NAD^+^ to form a tRNA with an ADP-ribosylated 2′-phosphate and release nicotinamide; and (ii) the 2″-hydroxyl of ribose of NAD^+^ nucleophilically attacks the 2′-phosphate to generate ADP-ribose 1″,2″ cyclic phosphate and tRNA with 2′-hydroxyl [48]. The overall structure of Tpt1 is similar to those of other ARTs, but the important residues H-Y-E are replaced by H-H-V. Generally, the last glutamate residue of H-Y-E is thought to be essential for the cleavage of NAD^+^ via the S_N_1 reaction. Therefore, it remains unclear how ParT and Tpt1 undertake the ADP-ribosylation reaction.

The human Deltex E3 ligases—DTX3L, DTX1, and DTX4—also catalyze the ADP-ribosylation of ubiquitin on Gly76 using the E2-ubiquitin thioester complex; this ADP-ribosylation does not exert a toxin function but rather contributes to the regulation of cellular processes via crosstalk between ADP-ribosylation and ubiquitination [49,50]. Deltex proteins have a conserved RING domain, which is characteristic of a RING-type E3 ligase and a Deltex carboxyl-terminal (DTC) domain in the C-terminus. Deltex forms a heterodimer with PARP9, which is a mono-ADP-ribosyltransferase reported to be enzymatically inactive, and Yang, C.-S. et al. reported that the heterodimer ADP-ribosylates the carboxyl group of ubiquitin Gly76, which is normally used for the conjugation of ubiquitin to protein substrates [49]. However, it was recently reported that this ADP-ribosylation is PARP9-independent, in that it requires only the RING and DTC domains of the Deltex protein [50]. Although the precise mechanism underlying this ADP-ribosylation remains unclear, the following mechanism is herein proposed: The E2-ubiquitin thioester complex binds to the RING domain, and the thioester bond is hydrolyzed to release ubiquitin. The carboxyl group of ubiquitin Gly76 nucleophilically attacks the NAD^+^ bound to the DTC domain to generate ubiquitin ADP-ribosylated on Gly76 and nicotinamide. Importantly, the structure of the DTC domain is not similar to those of the other ARTs, and the conformation of the bound NAD^+^ is also different from those bound to other ARTs. Thus, it seems that the DTC domain undertakes the ADP-ribosylation reaction via a totally different mechanism.

## 6. Substrate and Target Residue Recognition Mechanisms of R-S-E Class ARTs

The ability to specifically modify various residues is a key aspect of ARTs. For the R-S-E class ARTs, the ADP-ribosylating toxin turn-turn (ARTT) loop is thought to be important for substrate recognition [51]. The ARTT loop is composed of eight amino acids: X-X-ϕ-X-X-E/Q-X-E (Figure 1a). At the third amino acid position, the symbol ϕ refers to the presence of an aromatic residue (Phe, Trp, or Tyr); this residue is important for substrate recognition. The sixth amino acid is glutamate or glutamine and is important for both substrate recognition and target residue specificity. Ia has glutamate in this position and modifies arginine, while C3 has a glutamine in this position and modifies asparagine. Altering this glutamine to glutamate in C3 changes its specificity from asparagine to arginine [52]. Therefore, it has been suggested that the sixth amino acid (glutamate or glutamine) interacts with the target residue as follows: The carboxyl group of glutamate in Ia forms two hydrogen bonds with the guanidium group of the target arginine, while the carboxamide group of glutamine in C3 forms two hydrogen bonds with the carboxamide group of the target asparagine.

**Figure 2:**
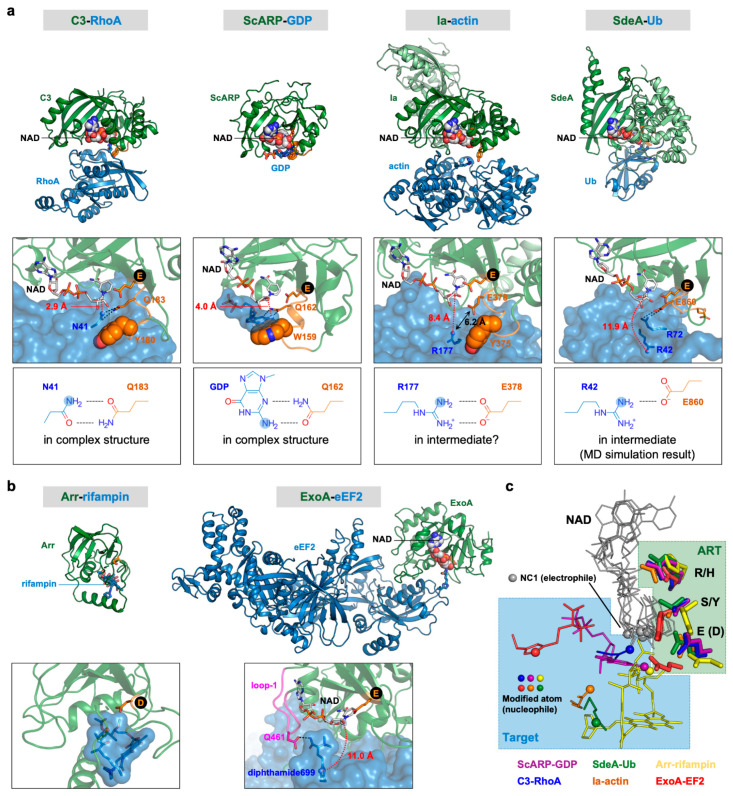
Comparison of complex structures of ARTs and their substrates. (**a**) R-S-E class ARTs. Top, overall structures; middle, close-up views of active sites; bottom, interactions between the target and the sixth amino acid of the ARTT loop. (**b**) H-Y-E class ARTs. Top, overall structures; bottom, close-up views of active sites. The N-terminal domain of Ia and the α-helical domain of SdeA are shown in light green color. The ARTT loops of R-S-E class ARTs are shown in orange. The NC1 of NAD (electrophile) and the modified atom (nucleophile) are shown as sphere models. Distances between two atoms involved in forming the new bond, namely the NC1 of NAD (electrophile) and the modified atom (nucleophile), are shown in red. Black circles indicate the third catalytic glutamate residues of the R-S-E and H-Y-E motifs. (**c**) Positional relationships between nucleophiles and NADs. Superimposition of six complex structures are shown in (**a**,**b**). R-S-E and H-Y-E residues are shown as stick models. NADs and targets are shown as lines. NC1 atoms of NADs (electrophiles) and modified atoms (nucleophile) are shown as sphere models. PDB IDs: Ia–actin, 4h03; C3–RhoA, 4xsh; SdeA–Ub, 5yij; ScARP–GDP, 5zj5; ExoA–eEF2, 2zit; Arr–rifampin, 2hw2.

We revealed the ternary complex structures of C3, RhoA, and NADH and confirmed the importance of the ARTT loop in substrate recognition and target residue specificity (Figure 2a) [53]. C3 family members have a tyrosine or phenylalanine in the ϕ position of the ARTT loop. C3cer, which we used in our crystallization, has tyrosine in this position; the tyrosine (Tyr180) side chain fits into a hydrophobic pocket formed by Val42, Ala56, and Trp58 of RhoA, verifying that Tyr180 plays in important role in the interaction with substrate RhoA. Importantly, the complex structure shows that Gln183, which is the sixth residue of the ARTT loop of C3, interacts with Asn41 of RhoA via two hydrogen bonds, as previously stated; this verifies the important role of this residue for target residue specificity. Moreover, the interaction arranges the nitrogen atom of the carboxamide group of Asn41 (nucleophile) at a position 2.9 Å distant from the NC1 carbon atom of NAD (electrophile), which is an appropriate position to attack the NC1 carbon atom and form a new bond. This was the first structure to verify the importance of the ARTT loop in substrate recognition and target residue specificity. Recently, we revealed the ternary complex structure of ScARP, GDP, and NADH (Figure 2a) [54]. Unlike C3, ScARP is a DNA-targeting ART; however, the interaction between ScARP and the substrate GDP via the ARTT loop is the same as that seen for C3. In ScARP, the flat indole ring of Trp159 in the ϕ position of the ARTT loop was found to form a π-stack interaction with the flat guanine ring of GDP, verifying that it plays an important role in the interaction with substrate GDP. Glutamine162 (the sixth residue of the ARTT loop) was found to interact with the guanidine moiety in the guanine ring via two hydrogen bonds, verifying its important role in target residue specificity. Moreover, this interaction arranges the nitrogen atom of the guanidine moiety (nucleophile) at a position 4.0 Å distant from the NC1 carbon atom of NAD (electrophile). This was the second structure to show the importance of ARTT loop for substrate recognition and target residue specificity. Interestingly, superposition of the two complex structures clearly shows that the positional relationship between ARTT loop, target atom, and NAD is the same in the protein- and DNA-targeting ARTs (Figure 2c).

We also revealed the ternary complex structures of Ia, actin, and NAD^+^ or β-TAD (an NAD analog) and the complex structure of Ia and ADP-ribosylated actin (Figure 2a) [55,56,57]. Ia homolog proteins have tyrosine or phenylalanine in the ϕ position of the ARTT loop. Ia has Tyr375 in this position, and the Tyr375 side chain fits into the hydrophobic pocket of actin consisting of Ile75, Leu110, Pro112, and Arg177 and forms a hydrogen bond with the carbonyl oxygen atom of the Leu110 main chain and thus plays an important role in substrate recognition. Unlike the situations in C3 and ScARP, Glu378 (the sixth residue of the ARTT loop) is positioned 6.2 Å from the side chain of target residue Arg177. Moreover, the modified nitrogen atom of Arg177 (nucleophile) is 8.4 Å away from the NC1 carbon atom of NAD (electrophile). Given that the target Arg177 nucleophilically attacks the NC1 carbon atom of NAD, the positional relationship of the ARTT loop, target Arg177, and NAD observed in the crystal structures is not appropriate for the reaction. Therefore, we proposed a strain-alleviation model wherein the reaction proceeds via three steps [56]: (i) The cleavage of NAD^+^, which binds to Ia in the strained conformation, occurs via the S_N_1 mechanism to release ADP-ribose with an oxocarbenium cation and nicotinamide; (ii) when the AMP moiety in ADP-ribose is gripped by Ia, the phosphoribose moiety in ADP-ribose moves close to the target Arg177 via bond rotation of pyrophosphate moiety, leading to alleviation of the ADP-ribose conformation—simultaneously, conformational changes of the target Arg177 of actin and Glu378 of Ia may induce an interaction between Arg177 and Glu378 via two hydrogen bonds (as observed in C3 and ScARP) and rearrange Arg177 to the appropriate position; and (iii) the nitrogen atom of Arg177 nucleophilically attacks the NC1 carbon atom of ADP-ribose and forms a new bond.

The ternary complex structures of SdeA–Ub–NADH and SidE–Ub^R42A^–NAD^+^ have been determined (Figure 2a) [58,59]. Because the amino acid sequences of the ART domains of SdeA and SidE show 72% identity to one another, it is not surprising that the complex structures show the same features. The ART domains of SdeA and SidE comprise an N-terminal α-helical lobe and a C-terminal ART-core lobe that shows similarity to other ART structures and has an R-S-E motif. NAD binds to the cleft formed between these two lobes. The ARTT loops (SdeA, 855-HGEGTESE-862; SidE, 850-HVSGSESE-857) are disordered in the absence of Ub. During SdeA–Ub or SidE–Ub complex formation, the C-terminal tail of Ub mediates most of the interactions with SdeA, especially the interactions with the ARTT loop and α-helical lobe, which cause the ARTT loop to become ordered. Surprisingly, the sixth residue of the ARTT loop, Glu860 in SdeA or Glu855 in SidE, do not interact with target residue Arg42 but rather form two hydrogen bonds with Arg72. In these structures, Arg42 is further away from NAD than Arg72. Dong Yanan et al. performed a molecular dynamics simulation in which the NADH of SdeA–Ub–NADH was replaced by ADP-ribose with an oxocarbenium cation [58]. This simulation showed that under this condition Arg72 moves away from the ADP-ribose moiety, and Arg42 (nucleophile) approaches to 4.46 Å from the NC1 carbon atom of the ADP-ribose moiety (electrophile) and forms an electrostatic interaction with Glu860 of the ARTT loop. However, the distance of 4.46 Å is still too far for nucleophilic attack. The relative position of Arg42 to NAD is similar to that of Arg177 of actin, suggesting that a conformational change via strain-alleviation may put the NC1 atom closer to Arg42 (Figure 2c). SdeA and SidE have glutamate and serine, respectively, in the ϕ position of the ARTT loop and thus lack an aromatic residue at this position. Thus, unlike the situations in C3, ScARP, and Ia, these residues do not contribute to the binding with Ub.

## 7. Substrate and Target Residue Recognition Mechanisms of H-Y-E Class ARTs

The ART core structures of the H-Y-E and R-S-E classes are similar; however, unlike the R-S-E class, members of the H-Y-E class do not have an ARTT loop, and any hypothesis on the substrate and target residue recognition mechanisms of this class have not been proposed. Two complex structures of H-Y-E ARTs and their substrate have been revealed to date, namely those of Arr and ExoA [40,60,61] (Figure 2b). The complex structure of Arr and its substrate antibiotic, rifampin, shows that they mainly interact via hydrophobic interactions. Although the complex structure with NAD is not available, superimposition of the complex structures for Arr–rifampin and ScARP–GDP–NADH shows that the H-Y-E motif of Arr and the nucleophilic oxygen atom of the rifampin hydroxyl group are located in spatially similar positions relative to the R-S-E motif of ScARP and the nucleophile nitrogen atom of GDP, respectively (Figure 2c). This suggests that the reactions of the H-Y-E and R-S-E classes proceed via a common S_N_1 mechanism. Another complex structure, ExoA–eEF2–NAD^+^, showed that the N3 atom of the imidazole ring of diphthamide (nucleophile) is 11.0 Å distant from the NC1 carbon atom of NAD (electrophile) [60,61]. Comparing the structures of ExoA–eEF2–NAD^+^ and ExoA–(ADPribosylated-eEF2) reveals that loop 1 of ExoA–(457-ARSQDLDA-464) is flexible. The conformation of loop 1 changes only in the NAD^+^-bound structure to form a hydrogen bond between Gln461 of loop 1 and diphthamide and to cap the diphthamide-binding pocket. In the transition state, it was proposed that diphthamide gets close enough to attack the electrophile [61]. It should be noted that this conformational change of the nucleophile contrasts with the electrophile conformation change seen in Ia. The positional relationship between diphthamide and NAD is different from that between NAD and Arg177 of actin, which is the target of Ia (Figure 2c): this may explain why the reaction mechanism is different.

PARP1 and PARP2 lack an ARTT loop and their structures show that they do not have a residue corresponding to the sixth residue of the ARTT loop. However, the accessory factor HPF1 binds to PARP1 and PARP2 and provides a glutamate residue that occupies a position equivalent to that of the sixth glutamate of the ARTT loop, leading to the formation of a composite active site [62]. Although the bindings of both HPF1 and NAD are sterically blocked by the α-helical domain of PARP, the binding of PARP to a dsDNA break induces a conformational change in the α-helical domain to enable the binding of both HPF1 and NAD [62,63]. The binding of HPF1 switches the specificity from aspartate or glutamate to serine [64]. Alanine substitution of the glutamate residue of HPF1 prevents serine ADP-ribosylation, suggesting that the glutamate from HPF1 determines the specificity for the serine residue, as seen in R-S-E class ARTs. However, it remains unclear whether the glutamate of HPF1 interacts with the target serine residue of its substrates, such as PARP1 and histone. In the future, analysis of the quaternary complex structure of PARP, HPF1, substrate, and NAD should provide deep insights into the functions of both H-Y-E and R-S-E class ARTs.

## 8. Conclusions

Recent reports revealing the complex structures of the protein-targeting ART, C3, and the DNA-targeting ART, ScARP, revealed that common mechanisms of target residue specificity are undertaken via the ARTT loop. However, the mechanisms underlying the specificities of TccC3, PT, and PARP for threonine, cysteine, and serine, respectively, remain unresolved. Moreover, the reaction mechanisms of ParT, DarT, and Tpt1 may be different, as these ARTs lack the key R-S-E or H-Y-E motifs. Going forward, efforts to determine the complex structures of ARTs and their substrates will provide important information and improve our understanding of the critical specificities of ARTs for their substrates and target residues.

## Data Availability

Data sharing is not applicable.
